# Compatibility of the CEN-ISO/TS 82304-2 Health App Assessment Framework With Catalan and Italian Health Authorities’ Needs: Qualitative Interview Study

**DOI:** 10.2196/67855

**Published:** 2025-04-21

**Authors:** Petra Hoogendoorn, Mariam Shokralla, Romy Willemsen, Nick Guldemond, María Villalobos-Quesada

**Affiliations:** 1 National eHealth Living Lab Public Health and Primary Care Department Leiden University Medical Center Leiden The Netherlands; 2 Research Center for Medical Sociology Tsinghua University Beijing China

**Keywords:** assessment frameworks, mobile health, mHealth, health apps, wellness apps, digital transformation, Italy, Catalonia, diffusion of innovations, value proposition canvas

## Abstract

**Background:**

Health authorities of European Union (EU) member states are increasingly working to integrate quality health apps into their health care systems. Given the current lack of unified EU assessment criteria, the European Commission initiated Technical Specification (TS) CEN-ISO 82304-2:2021*—*Health and wellness apps*—*Quality and reliability (hereinafter the “TS”) to address the scattered EU landscape of assessment frameworks (AFs) for health apps. The adoption of an AF, such as the TS, falls within member state competence and is considered an uncertainty-reduction process. Evaluations by peers as well as ensuring the compatibility of the TS with the needs of health authorities can reduce uncertainty and mediate harmonization.

**Objective:**

This study aims to examine the compatibility of the TS with the needs of Catalan and Italian health authorities.

**Methods:**

Semistructured interviews were conducted with key informants from a regional (Catalonia in Spain) and national (Italy) health authority, and a thematic analysis was carried out. Main themes were established deductively, following the aspects defined by the value proposition canvas: (1) health authorities’ needs (“gains,” “pains,” and “jobs”) and (2) the TS “products and services” and their distinct characteristics (“gain creators” and “pain relievers”). Subthemes were generated inductively. The compatibility of the needs with the TS was theoretically determined by the researchers. The results were visualized using the value proposition canvas. Two participant validation steps confirmed that the most relevant aspects of the predefined themes had been captured.

**Results:**

Despite the diversity of the 2 health authorities, subthemes were common and categorized into 9 gains, 9 pains, and 11 jobs. Key findings include the health authorities’ perceived value of, and need for, integrating quality health apps and using an AF (gains), along with the related policy, implementation, and operational activities (jobs). The lack of enabling EU legislation and standardization, resulting in a need for the multiple authorities involved to consent, made achieving an AF challenging (pains). Nine products and services related to the TS and 17 distinct characteristics (eg, its multistakeholder evidence base) were found to be compatible with 3 gains (eg, stimulating the prescription and use of apps), 7 pains (eg, legislation and harmonization issues), and 6 jobs (eg, assessing apps). Indirect effects, 3 anticipated future services, and 1 anticipated gain creator and pain reliever increase this compatibility.

**Conclusions:**

Our results suggest that the health authorities share common fundamental needs, and that the TS is compatible with these needs. The identified needs and compatibility can potentially reduce peer authorities’ uncertainties in adopting an AF in general and the TS in particular. More research is recommended to confirm and translate our results in other contexts and further fine-tune compatibility to achieve wide adoption of the TS and accelerate the uptake of health apps.

## Introduction

### Background

Health authorities of European Union (EU) member states are transforming health and care systems to address current challenges and remove cross-border regulatory barriers for businesses and consumers to progress toward an EU digital single market [1-6]. In this context, health apps (Textbox 1) are gaining attention, and countries are adapting their policies and structures to harvest the potential of these digital solutions to strengthen their health care systems [5,7-10].

**Definitions of health, medical, and wellness apps.**

In the context of this paper, health apps are defined as apps that are “intended to be used specifically for managing, maintaining or improving health of individual persons or the delivery of care” [11]. Health apps are part of mobile health, which is defined as “medical and public health practice supported by mobile devices, such as mobile phones, patient monitoring devices, personal digital assistants (PDAs), and other wireless devices” [12]. Health apps include medical apps and wellness apps. Medical apps have been defined as those that fall under an applicable medical device regulation and wellness apps as those that do not [13].

Not all apps are of good quality, that is, have a positive and reliable effect on health, and are easy to use, compliant with privacy and data security regulations and standards, robust, and interoperable with electronic health record systems [8,9,14]. Determining the quality of individual health apps is challenging for citizens, health care professionals (HCPs), and decision makers [15] for various reasons. First, assessing apps requires specific and diverse expertise. Second, robust evidence (eg, clinical evidence) and background information are often scarce, inappropriate, or not publicly available [14]. Third, quality assessments and widely adopted evidence-based evaluation methodologies that consider all these aspects and inform (potential) users about the quality of these apps are not yet common [10].

Several health authorities have developed their own assessment frameworks (AFs) [16]. However, their efficient implementation is challenging [7,15]. The significant overlap in quality criteria across these AFs highlights the potential for harmonization and related efficiency [17]. In addition, nonoverlapping quality criteria—a lack of agreement on what constitutes quality—potentially decrease trust [17] and increase inequality (eg, if the challenges to comply with the different AFs result in manufacturers focusing only on the larger markets and widely spoken languages). In the context of multilingualism—an EU founding principle [18]—and an abundance of health apps worldwide [19], the availability of native language health apps for the 5 million Finnish-speaking EU residents, for instance, is limited [20]. These aspects highlight the need for the harmonization of AFs at an EU level.

Both academics and policy makers endorse harmonization and cross-national regulation of health apps to realize their full potential and benefits [7,10]. The European Commission initiated CEN-ISO/TS (Comité Européen de Normalisation [European Committee for Standardization]–International Organization for Standardization/Technical Specification) 82304-2:2021 Health software*—*Part 2: Health and wellness apps*—*Quality and reliability (hereinafter the “TS”; Textbox 2) to address the scattered EU landscape of health app AFs and progress from 27 national and even more regional markets to a digital single market [6,11]. To achieve EU-wide harmonization, the adoption of the TS among EU member states is needed, which falls within member state competence and responsibility. Adoption across member states can be described as the diffusion of the TS as an innovation.

**Brief description of CEN-ISO/TS (Comité Européen de Normalisation [European Committee for Standardization]–International Organization for Standardization/Technical Specification) 82304-2:2021 Health software—Part 2: Health and wellness apps—Quality and reliability (the TS).**

CEN-ISO/TS 82304-2:2021 was an assignment from the European Commission to CEN that became a global effort in its collaboration with ISO and the International Electrotechnical Commission (IEC). The TS has 2 core products. The first is a health app assessment framework (AF) developed through a Delphi study with 83 experts [21]. The second is a quality label (Multimedia Appendix 1) that was inspired by the European Union (EU) energy label and Nutri-Score front-of-pack nutrition label designs and the US Food and Drug Administration over-the-counter medicine label content. The quality label allows the easy and accessible presentation of the assessment results of a health app and was developed and tested with people with low health literacy. The EU-wide implementation readiness of the TS was supported by the Horizon Europe Label2Enable project (2022-2024), which tested the quality label and tested and developed products and services complementary to the AF and quality label [22,23]. These include the health app quality report, which is the detailed version of the label that aims to support health care professionals in their decision-making on recommending health apps; the certification scheme, which includes the handbook for app assessment with CEN-ISO/TS 82304-2 for certified app assessment organizations; stakeholder guidance and tools such as educational videos; and recommendations for the reimbursement of health apps. A multistakeholder road map was generated to guide next steps [24].

Innovations and their adoption process can be understood using the theory of diffusion of innovations proposed by Rogers [25], who identified 3 main attributes of an innovation that predict its future rate of adoption. Compatibility is one of these attributes and refers to the degree to which an innovation is perceived as consistent with the existing values, experiences, and needs (summarized hereinafter as “needs”) of potential adopters. The diffusion of an innovation is considered an uncertainty-reduction process. An innovation that is compatible generates less uncertainty for the potential adopter regarding the success of its deployment. The other main attributes that predict the future rate of adoption—relative advantage and complexity—are beyond the scope of this paper and will be analyzed in separate studies.

### Objectives

Potential adopters tend to rely on peers for information about an innovation’s desirable, direct, and anticipated consequences to decrease their uncertainty about adopting it [25]. In this paper, we aim to examine the compatibility of the TS with the needs of 2 health authorities in the EU, in order to potentially enhance the compatibility of the TS and reduce the uncertainty of peer health authorities in their considerations on adopting the TS.

## Methods

### Overview

**Figure 1:**
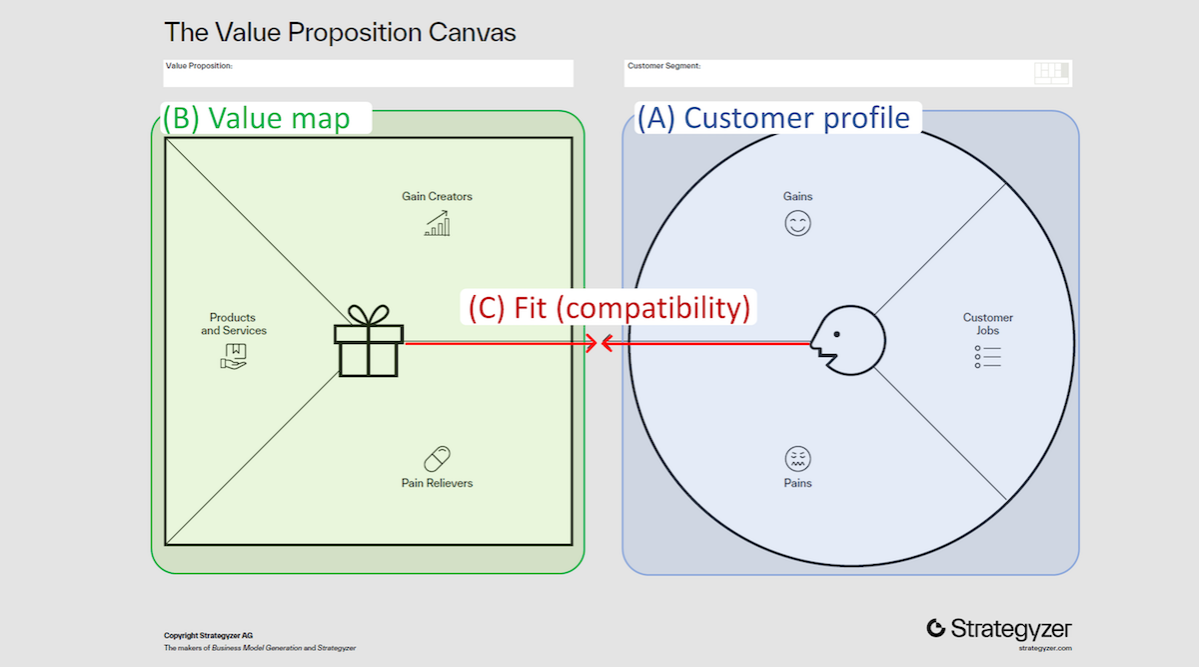
The template of the value proposition canvas [29]. Adapted from Strategyzer [33] to highlight the 3 main steps in constructing the value proposition canvas (Textbox 3): (A) the customer profile with the customer needs (jobs, gains, and pains), (B) the value map with the innovations’ products and services and their distinct characteristics (gain creators and pain relievers) that aim to address the customer needs, and (C) the fit (compatibility) of the customer profile with the value map.

Rogers [25] described different methods to research the attributes of innovations, including compatibility. For this study, investigating an innovation’s acceptability in its prediffusion stage (eg, test-marketing and piloting) was considered the most appropriate approach. This approach helps identify a “basis for positioning an innovation so that it will be more acceptable, that is, have a more rapid rate of adoption” [25]. Key informant interviews are a common method for assessing the acceptability of an intervention [26,27]. Key informants provide high-level perspectives and comparative insights. Their particular roles and expertise make them especially equipped for probing about how a topic is thought about or acted upon in policy. This contrasts with the scope of insight contributed by other groups of qualitative interviewees, who are more often recruited to provide data rooted in their own lived experiences, opinions, and beliefs [27]. A widely used business tool to visualize and enhance the compatibility (also referred to as “fit”) of innovations is the value proposition canvas (VPC; Figure 1; Textbox 3) [28,29]. The VPC tool has been previously applied to guide the development of, among others, digital health innovations [30-32].

**Glossary of value proposition canvas concepts.**

- The customer profile (Figure 1A) describes a potential adopter segment, in this case health authorities, in terms of their needs:
  Jobs—the tasks or responsibilities potential adopters have, need, or want to get done in their work and their lives, in this case with respect to adopting an assessment framework and integrating health apps in their health care system
  Gains—outcomes and benefits potential adopters require, expect, or desire to achieve if these jobs are done (well)
  Pains—negative outcomes, risks, and obstacles that adopters encounter, foresee, or fear that prevent them from getting these jobs done (well)
- The value map (Figure 1B) makes explicit how the innovation creates value for the potential adopters’ segment:
  Products and services—the range of products and services available to the potential adopters to address their needs, in this case with respect to adopting an assessment framework and integrating health apps
  Gain creators—the characteristics of these products and services that generate gains for the adopters
  Pain relievers—the characteristics of these products and services that alleviate the adopters’ pains
- Fit (Figure 1C): when the products and services, gain creators and pain relievers contribute to realizing the jobs and gains, and addressing the pains of the potential adopters ("compatibility")

In this paper, we used the VPC to visualize the compatibility of the TS with the needs of 2 health authorities (potential adopters of the TS):

1. A regional authority—Fundació TIC Salut Social (FTSS; TIC Salut Social Foundation), the public organization that oversees digital transformation in health care in Catalonia, Spain
2. A national authority—Italy’s Istituto Superiore di Sanità (ISS; National Institute of Health)

Catalonia established its own AF in 2017 [34-36] and is pursuing the adoption of the TS [37]. Italy drafted a Technical Report about wellness, social, and health apps, which informed the TS [38], and has been using the TS as inspiration in the ongoing process to arrive at quality assessment criteria for the inclusion of telehealth solutions in a national telehealth catalog, where telehealth includes health apps. FTSS and ISS jointly represent the health authority perspective in the Label2Enable project (Textbox 2) and were recruited for this study. Recruitment criteria spanned experience as an EU-based health authority with the relatively novel integration of health apps, prediffusion interest in a standardized AF, and diversity (country or region, number of inhabitants, and an AF in place or in development). These were considered essential for providing the rich basis for analyzing the TS in terms of prediffusion acceptability or compatibility [25].

### Key Informants

Three key informants were recruited. They were chosen strategically, in alignment with existing knowledge of the qualities of “good” key informants [27]. Eligibility criteria included a key leading position in their respective organizations over the course of several years in our topic of interest and related inside in-depth knowledge about the authorities’ policy and plans to assess health apps as well as execution of these plans. For FTSS, the key informant has been responsible for implementing Catalonia’s mobile health (mHealth) plan in FTSS for the past 9 years. For ISS, the first key informant had recently retired from a leadership position at ISS, which involved advising the government about health apps and telemonitoring. The second ISS key informant was recruited after discussion with the first ISS key informant, based on complementary first-hand experience on the topic in dealings with the Italian Ministry of Health. The 2 ISS informants played a direct role in drafting the Technical Report, which informed the TS [38]. While the number of potential participants can be very large in other types of qualitative research, key informant groups can be quite small, hard to reach, or constrained in sharing information with researchers [27].

Key informants and study researchers collaborated in the Label2Enable project; however, the key informants were not otherwise involved in these research activities. The researchers who conducted the interviews and analysis were experienced in qualitative, mixed methods, and implementation research in the field of digital health. Interest in the research topic stems from their previous work related to health apps, other digital health technologies, digital transformation, and the TS.

### Constructing the VPC

Constructing the VPC involves 3 steps (Figure 1; Textbox 3) [29], which we carried out systematically using qualitative research methods. The first step was to develop the “customer profile” (Figure 1A). Here, the needs of the health authorities (potential adopters of the TS) were described in terms of the 2 authorities’ “gains,” “jobs,” and “pains” (Textbox 3). Strategyzer’s “trigger questions” served as a guide for designing the semistructured interviews (Multimedia Appendix 2) [33].

Two semistructured interviews (one for Catalonia and one for Italy) were conducted, in English, with the relevant key informants. The semistructured interview questions were shared with the key informants in advance. The interviews were conducted on the web, recorded using a secure digital platform, and lasted between 1.5 and 2 hours. Recordings were transcribed verbatim and coded independently by 2 researchers using ATLAS.ti software (version 7.9; ATLAS.ti Scientific Software Development GmbH). A deductive and inductive thematic analysis of the interviews was performed. On the basis of the VPC, we predefined 3 main themes: gains, pains, and jobs [29]. We classified a job as a past, current, or future task or responsibility of a health authority related to adopting an AF to integrate health apps into their health care system. Jobs could be the responsibility of the health authority featured in this study or associated health authorities because, from an international perspective, health authorities’ task divisions may differ and evolve. We applied the same principle to the gains and pains. Subthemes were inductively developed by 2 researchers and visualized using the VPC.

Data saturation was not deemed appropriate for this study; instead, inductive thematic saturation [39] was sought across and within cases to achieve a “basis for positioning the innovation [the TS] so that it will be more acceptable,” knowing that uncertainty is inherent in the prediffusion phase, and that adopting organizations will adapt an innovation to achieve a fit of the innovation with the adopting organization and its perceived problem [25]. Accounting for data sufficiency is an underdeveloped aspect of existing methodological guidance on key informant sampling [27]. Incorporating strategies to return the findings and elicit key informant response on researcher interpretations is recommended to yield rich insights [27]. With this in mind, a participant validation step that lasted between 30 and 60 minutes was carried out separately per health authority. Key informants were supplied with the written data analysis before and after the validation step, allowing them to provide comments and enrich the analysis. The key informants confirmed that the researchers had captured the most relevant aspects within the 3 predefined main themes: gains, pains, and jobs [28].

The second step was to develop the “value map” (Figure 1B), which consists of describing the innovation’s “products and services,” “pain relievers,” and “gain creators” (Textbox 3). Outputs from all Label2Enable work packages were considered [22,23]. Products and services included those that are currently part of the TS, those that were cocreated in the Label2Enable project (both Textbox 2), and products and services that will be generated beyond the project during the “demonstrator phase.” The relevant characteristics of these products and services were subsequently added to the value map as gain creators and pain relievers.

The third step was to determine the fit (Figure 1C), that is, to check whether the “value map” was compatible with the “customer profile.” In other words, whether the TS and the related products and services and their distinct characteristics (gain creators and pain relievers) satisfied the needs (jobs, gains, and pains) of the health authorities. Visualizing compatibility (fit) was an iterative process of determining and conceptualizing the products and services, gain creators, and pain relievers that related to the health authorities’ gains, pains, and jobs. After completing this process, a second participant validation step, which lasted approximately 1 hour, was carried out with the key informants from both health authorities simultaneously. Again, the key informants were supplied with the written data analysis before and after the validation step, allowing them to provide comments and enrich the analysis. Furthermore, the manuscript was discussed with the key informants at different stages, and they confirmed that it condensed the most relevant aspects surrounding the position of their institutions and agreed to its publication in its current form.

Data were reported according to the COREQ (Consolidated Criteria for Reporting Qualitative Research) checklist (Multimedia Appendix 3).

### Ethical Considerations

Approval from an ethics or scientific research committee was not required under Dutch national regulations because the study did not fall under the Medical Research Involving Human Subjects Act (WMO), and according to the guidelines of the Central Committee on Research Involving Human Subjects (CCMO [40]). This study followed institutional good research practices and integrity codes. Verbal consent to conduct and record the interviews was obtained from all participants (key informants) before starting the interview. Personal data were protected in accordance with European Union and institutional standards and best practices. The 2 organizations represented were funded as Label2Enable project partners and were not additionally compensated for their participation in this study.

## Results

### Customer Profile

**Figure 2:**
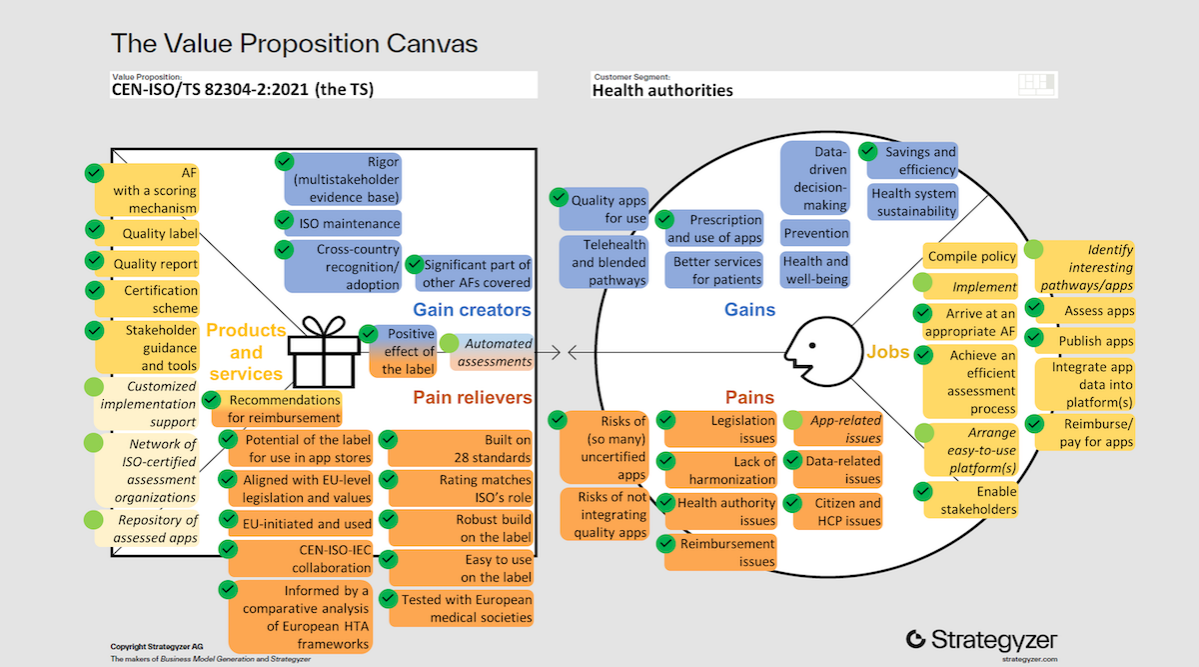
Adapted from Strategyzer [33], the value proposition canvas of CEN-ISO/TS (Comité Européen de Normalisation [European Committee for Standardization]–International Organization for Standardization/Technical Specification) 82304-2:2021 Health software—Part 2: Health and wellness apps—Quality and reliability (the TS) for health authorities pursuing an assessment framework (AF) to integrate health apps into their health care systems. The “customer profile” (Figure 1A) summarizing the Catalan and Italian health authorities’ needs (gains, pains, and jobs) resulting from the deductive and inductive thematic analysis. The “value map” (Figure 1B) of the TS, with the products and services related to the TS and their distinct characteristics (gain creators and pain relievers) that aim to address these health authorities’ needs as identified by the researchers. Lighter colored boxes with italic text indicate future services, gain creators, and pain relievers. Dark green circles with a check mark indicate the fit (compatibility; Figure 1C) of a health authority’s need with an existing product or service, gain creator, or pain reliever. Light green circles without a check mark and italic text indicate expected future fit (compatibility) derived from the nature of the anticipated related services and gain creator or pain reliever. The tables in this paper show the specific products, services, or characteristics that fit specific customer needs (gain, pain, or job). AF: assessment framework; EU: European Union; HCP: health care professional; HTA: health technology assessment; IEC: International Electrotechnical Commission.

In this subsection, we describe the needs of health authorities (potential adopters) in terms of gains, pains, and jobs. Despite the diversity of the 2 health authorities, subthemes were common and categorized into 9 gains, 9 pains, and 11 jobs (Figure 2).

#### Gains

Regarding the gains—the outcomes and benefits that the health authorities require, expect, or desire to achieve from adopting an AF and integrating quality health apps—the key informants explained the interest of their region (Catalonia, Spain) or country (Italy) in an AF to identify “quality apps for use” in “telehealth (Italy) and blended pathways (Catalonia)" (Textbox 4).

**Key definitions in the Catalonian and Italian context.**

**Blended care in Catalonia**

- “Blended care” “refers to an integration of online and offline components in a treatment process. This means that online and offline components are interconnected in some way and not stand-alone treatment pathways [41]. In Catalonia, according to the mobile health plan, mobile health should have an impact on the full “cycle of provision of care” (prevention, diagnostics, treatment, and follow-up) and ultimately on health, the health care system, and public health. In addition, it prioritizes the 10 most prevalent diseases (eg, diabetes) [42].

**Telehealth in Italy**

- The World Health Organization defines telehealth as the “delivery of health care services, where patients and providers are separated by distance” [12]. In the Italian context, telehealth or telemedicine is an innovative approach to health care practice that enables the remote provision of services through the use of digital devices, the internet, software, and telecommunication networks. Telehealth services can serve as an alternative to traditional care, support existing services (eg, by improving accessibility, efficiency, and equity), supplement care (eg, by improving effectiveness and facilitating personalized medicine), or completely replace traditional services [43].

The key informants described how the assessment of apps is instrumental to achieving the “prescription and use of apps,” referring to incorporating the prescription or recommendation of apps in the provision of care and the related trusted use of health apps by citizens. Similarly, it can contribute to “better services for patients” (eg, improved accessibility, lower waiting times, and better follow-up):

If you want these applications to be recommended by health care professionals, we need to provide safe and guaranteed apps to the sick. So, we try to establish how we can measure or evaluate these apps and the quality of these apps.

Key informant, Catalonia

Certainly, the availability of an agreed, evidence-based assessment framework will give objectivity to the activity of app assessment.

Key informant, Italy

They [patients] will have the opportunity to make more virtual visits, sharing the data with their professionals without needing to go to the consult, provide more alerts and provide more predictions, and prevention to avoid disease or avoid being sicker. Also, they will feel more confident and committed to healthier lifestyle habits when using wellness apps. And they can also be more guarded by their professionals. They will feel more followed up.

Key informant, Catalonia

Moreover, such apps can also streamline the access of the citizens to the NHS, for example, by cutting waiting times for a given service.

Key informant, Italy

For the key informants, the prescription and use of apps would enable “data-driven decision-making”—the use of health data derived from an individual’s health app use in the delivery of health care—seeing both benefits for the prescribing HCP and the provision of effective transversal care. Other benefits mentioned included the possibilities for “prevention,” through the promotion of healthier behaviors and end-user empowerment, and ultimately “health and well-being”:

To provide more autonomy to patients and to be more committed to their health, to keep tracking health data more efficiently and having all the information integrated into the public single network, so that all health care professionals are able to follow up the patient through the information system.

Key informant, Catalonia

The interest of our country is given by the opportunity of providing the best health status of the population, even with limited resources. Health apps can support primary and secondary prevention, by modifying lifestyles.

Key informant, Italy

The key informants described how all aforementioned gains translate into “savings and efficiency” and “health system sustainability,” mentioning aspects such as fewer in-person visits, more web-based visits, more equity, earlier diagnosis, and cross-country recognition of app assessments:

I think for health care professionals, they [apps] will reduce the number of face-to-face consults for more virtual [visits]. They will have a unique platform or a unique space to monitor the data of the patients...they receive alerts, for example, so that they focus their attention on critical issues, not revising a lot of data.

Key informant, Catalonia

My personal feeling for sure, savings at the health system level. Access for all the people, or the individuals, as my Constitution says.

Key informant, Italy

Another step that has to be done is updating the current certification framework and consider the possibility to adapt this framework to the Technical Specification of 82304-2 in order to improve the framework for cross-recognition.

Key informant, Catalonia

A sound evaluation framework, recognized by the EU, will be instrumental in optimizing the resources spent in assessing health apps in the different EU countries by means of the foreseeable cross-country validity of the approval.

Key informant, Italy

#### Pains

Regarding the pains—the negative outcomes, risks, and obstacles that health authorities face with respect to assessing and integrating apps—the key informants from both authorities mentioned challenges related to context, implementation, and app use. The “risks of (so many) uncertified apps” referred to the currently unknown quality of apps and the negative effects for patients, HCPs, and the health care system. At the same time, the key informants considered the “risks of not integrating quality apps,” emphasizing that not integrating apps would be an obstacle to providing better care and other important gains:

That is the main risk, not being adopted because professionals don’t feel that it helps. Because I think that the patient part is solved. They are using it a lot and they want to be involved.

Key informant, Catalonia

We’ve in Italy started a long time ago working on apps because we have realized that digital support to health care is absolutely mandatory, and it was clear that there was a sort of Wild Wild West with respect to the apps.

Key informant, Italy

The main risk, I think, will be not adopting mobile apps into the health care system, not providing sufficiently optimized care to the citizens compared to other territories.

Key informant, Catalonia

At present you lose data, you lose information, you lose the capability to cost, to build a new best practice because you have no sufficient critical mass of data to say something, also to use artificial intelligence appropriately.

Key informant, Italy

Implementation-related pains mentioned by the key informants included “legislation issues,” such as the lack of comprehensive legislation for apps that are not medical devices and the multiple applicable (emerging) EU legislations, which pose significant challenges to app developers and authorities. “Lack of harmonization” is a missed opportunity to alleviate the burden of legislation issues and potentially adds to the difficulties:

We are regulating medical devices, the apps that are also medical devices, thanks to the new regulation of medical devices, but we cannot regulate the apps that are not medical devices. That is why we are interested in integrating a sort of certification scheme similar to, or directly, the ISO (82304-2) Technical Specification.

Key informant, Italy

I am afraid of too much legislation. Too much vertical [legislation] that is not correlated to each other.

Key informant, Italy

We faced the challenge to create the framework from scratch because we did not have anything at the time.

Key informant, Catalonia

The risk in the immediate future is that some regional health system starts piloting an evaluation framework without national coordination, in that case...it would be more difficult to harmonize the assessment at national level, which could result in enhanced health inequalities.

Key informant, Italy

For the key informants, legislation issues and the lack of harmonization resulted in “health authority issues.” The integration of health apps into health care currently involves multiple authorities, whose interests, roles, and responsibilities related to health apps can be unclear, overlapping or conflicting. In addition, health apps present an uncharted territory and, may represent just one of these authorities’ many topics. This makes reaching agreements on issues such as an AF complex. In addition, publishing information about the quality of products without being able to refer to a recognized (international) standard could conflict with the authority’s role and be perceived as biased from the perspective of health app providers. Finally, the key informants refer to “reimbursement issues,” challenges related to structuring the reimbursement of health apps:

Something that is really difficult, and I think the European region has that problem, is the different actors to be aligned...Pff, a lot of actors, that each of them has their needs and it’s very difficult to get an agreement to move forward.

Key informant, Catalonia

We have too many agencies, too many institutions that manage all these definitions of digital health and digital health technologies [not aligned taxonomies]. That is why I hope that main documents from Europe could help us to be stricter in the field.

Key informant, Italy

Communicating the quality of a particular product, could actually lead to a “market bias,” that is, putting a product in a favorable position with respect to its competitors, although involuntarily, needless to say. We are instead interested in communicating the relevance of the framework for the app evaluation, as an objective and independent instrument for orienting health choices for personalized digital medicine.

Key informant, Italy

And after that comes defining the reimbursement models, well, I think it will take a lot [of time].

Key informant, Catalonia

The key informants identified several pains related to the identification, assessment, integration, recommendation, and use of health apps. These included “app-related issues” (eg, constant updates and a lack of clinical evidence), “data-related issues” (eg, not all data generated by apps are clinically relevant, with relevance generally differing per pathology and care pathway; moreover, there can be interoperability issues), and “citizen and HCP issues” (eg, limited digital skills):

Something that is very difficult is how you revalidate based on the framework, how do you reassess the apps. Because the apps change a lot.

Key informant, Catalonia

“At present what is lacking is clear sound evidence from clinical trials about the apps. This is a very great problem because also our Ministry of Health is waiting for clinical trial data for authorization of medical device apps and then to have the data from them.

Key informant, Italy

Then you [HCP] want to monitor a patient based on these health apps, and you want to integrate the data...It was very challenging because we had to find specific professionals for each pathology. They tell us or tell the group which are the relevant data.

Key informant, Catalonia

Improving the digital skills of professionals also is very challenging and we try to solve it as well, by providing more training.

Key informant, Catalonia

The target population of some interventions — for example, for frailty/chronicity — may find itself hardly capable of exploiting the technology, if the latter is not properly introduced, with a training appropriate for the digital skills of the subject.

Key informant, Italy

#### Jobs

Regarding the jobs—the tasks or responsibilities of health authorities in assessing and integrating apps—the key informants described activities related to policy, implementation, and operations. In Catalonia, “compile policy” referred mainly to the “mHealth plan” [42], which in 2015 triggered the appointment of FTSS to “implement” this plan and create an AF. In Italy, this job was linked to the recovery and resilience funding coordinated by the European Commission, which pushed plans for telehealth and an adequate AF [44]. The key informants discussed their roles as experts and advisers to the government and in the decision-making and implementation of an AF:

TIC Salut mHealth Office was established after publishing this agreement in 2015, an mHealth plan that was published or was agreed upon in the parliament, where they created TIC Salut in the mHealth area as an instrument to provide or to create these actions, to implement mHealth in the health sector.

Key informant, Catalonia

The National Resilience Program has given us quite a lot of support in that, and a lot of money has actually been poured into health care...One of the areas which has been impacted very much is telehealth, which includes health apps.

Key informant, Italy

Although the jobs overlap, the exact scope targeted in their policies and strategies differs. Catalonia emphasizes apps embedded in blended care, whereas Italy focuses on telehealth solutions:

The electronic health record of the patient was integrated, and we also had the personal portal for citizens. So, the gap that we did have was to integrate the data that came from apps.

Key informant, Catalonia

And so, the focus here in Italy at the moment is on telehealth solutions, which comprise health apps.

Key informant, Italy

Important steps in the implementation were, according to the key informants, to “arrive at an appropriate AF”; “achieve an efficient assessment process” that is moreover scalable; “arrange easy-to-use platform(s)”; and “enable stakeholders” by, for example, issuing recommendations, accessible information and tools (eg, self-assessment tools [45]):

So, those were the 2 big projects. One is the assessment framework and the other the mHealth platform that goes in parallel...The objective of this platform is to include apps that have passed this accreditation and to help health care professionals prescribe the apps to their patients.

Key informant, Catalonia

So, the ministry studied all the proposals from the different experts, and they actually decided to use 82304-2 as the inspirational assessment framework to define the assessment framework for telehealth solutions that are going to be onboarded onto this national catalog.

Key informant, Italy

The main objective is now the number of apps that we want to share. It’s our expectation to have at least 30 apps, or 50 depending. But this year at least try to get these 30.

Key informant, Catalonia

And also, we did recommendations, guides, tools. Also, we have the auto-evaluation on our website, that is a questionnaire free and open...We made it for the industry, for them being prepared, and to promote creating these quality apps.

Key informant, Catalonia

Operations start with what key informants refer to as a prioritization of the apps to integrate into the health care system (“identify interesting pathways/apps”):

So, we focus on diabetes because it’s something that we have different apps that are provided by private companies, and there are a lot of people with this disease, and it is expensive and it’s a priority for the health care system to provide tools in that way.

Key informant, Catalonia

So, there will be a pathway for diabetics, so there would be one for oncology, certain types of oncology and so on.

Key informant, Italy

We have these 2000 apps that are identified as interesting apps, that are not certified using our framework obviously, but they are being used by health care centers or they are being published in other frameworks like Andalusia or mHealth Belgium, et cetera.

Key informant, Catalonia

After this prioritization of apps that are interesting to assess, the assessment can be carried out (“assess apps”), and positively assessed apps and their results in specific quality domains and criteria can be shared (“publish apps”). The publication of assessed apps enables the series of gains, which starts with “quality apps for use.” The next operational step, to “integrate app data into platform(s),” enables the desired gain “data-driven decision-making.” Catalonia expects the capability to integrate app data into platforms to become available in 2025 for 1 care pathway, while Italy does not yet carry out assessments, and the division of responsibilities among Italian authorities is not yet defined.

For both health authorities featured in this study, the final step in the process is to “reimburse/pay for apps”:

Once these apps are validated, we publish the apps on our website, so all citizens know all aspects of the [certification] process.

Key informant, Catalonia

And in the next stage, we are expecting to integrate the data from these apps of diabetes in the platform that we create.

Key informant, Catalonia

We have a group that is working on a specific reimbursement model.

Key informant, Catalonia

A policy is at present under discussion thanks to a specific government workgroup on digital therapies that involves several stakeholders, scientific and industrial. The latter (drug and medical device industry organizations) are discussing a unitary proposal for a unique reimbursement scheme.

Key informant, Italy

### Value Map and Fit

#### Overview

In this subsection, we present the results of the iterative process of identifying the TS-related products and services and their distinct characteristics in relation to the needs of the health authorities. The needs are visualized in the VPC as the pains, gains, and jobs of the 2 health authorities. When a TS-related product or service or one of its characteristics (gain creator and pain reliever) helps to address or to achieve a gain, pain, or job of the health authorities, we considered it a “fit” (compatibility). The fit was visualized with a green circle (Figure 2). We found that the TS addressed 3 (33%) of 9 gains, 7 (78%) of 9 pains, and 6 (55%) of 11 jobs in part or in full. An anticipated gain creator and pain reliever and 3 TS-related anticipated future services would target 1 extra pain and 3 extra jobs, enhancing the compatibility to 3 (33%) of 9 gains, 8 (89%) of 9 pains, and 9 (82%) of 11 jobs. The 6 gains and 1 pain that remain would be influenced indirectly (eg, the TS supports the gain “quality apps for use,” which is a prerequisite for all further gains). This would leave only 2 jobs unaddressed: “compile policy” and “integrate app data into platform(s).”

#### Products and Services

In total, 6 current and 3 future products and services were identified, which are compatible with (“fit”) 9 jobs (Figure 2; Table 1). The 2 core products of the TS are an “AF with a scoring mechanism” and a health app “quality label” (Multimedia Appendix 1) [11]. The quality label is a key aspect that differentiates the TS from other AFs. In the Label2Enable project, four TS-related products were cocreated with the relevant stakeholders: (1) the health app “quality report,” which is a more detailed version of the quality label [22]; (2) the ISO 17000 series–compliant “certification scheme” for the TS, which includes the app assessment handbook for CEN-ISO/TS 82304-2 for certified conformity assessment bodies [22]; (3) “stakeholder guidance and tools,” which refers, for example, to educational videos [46] on the quality label for citizens and guidance for manufacturers; and (4) a series of “recommendations for reimbursement” of health apps [47]. The upcoming demonstrator phase aims to assess the first 100 apps, test and optimize the value of the health app quality report from a multistakeholder perspective and explore how to exploit the potential of “automated assessments” (refer to the next subsection, Gain Creators). This phase is planned to deliver 3 extra services: (1) “customized implementation support,” (2) a “network of ISO-certified assessment organizations,” and (3) a “repository of assessed apps.”

**Table 1:** Compatibility (or fit) of the jobs related to adopting an assessment framework (AF) for health apps as described by the Catalan and Italian health authorities and the products and services related to CEN-ISO/TS (Comité Européen de Normalisation [European Committee for Standardization]–International Organization for Standardization/Technical Specification) 82304-2:2021 Health software—Part 2: Health and wellness apps—Quality and reliability (the TS).

Jobs	Products and services
Implement^a^	Customized implementation support^a^
Arrive at an appropriate AF	AF with a scoring mechanism
Achieve an efficient assessment process	Certification scheme Network of ISO-certified assessment organizations^a^
Arrange easy-to-use platform(s)^a^	Repository of assessed apps^a^
Enable stakeholders	Stakeholder guidance and tools Network of ISO-certified assessment organizations^a^
Identify interesting pathways/apps^a^	Repository of assessed apps^a^
Assess apps	AF with a scoring mechanism Certification scheme Stakeholder guidance and tools Network of ISO-certified assessment organizations^a^
Publish apps	Quality label Quality report Repository of assessed apps^a^
Reimburse/pay for apps	Quality report Recommendations for reimbursement

^a^Job to be facilitated and corresponding anticipated TS service planned to be established during the demonstrator phase.

#### Gain Creators

Five current and one anticipated gain creators were identified (Figure 2), which are compatible with (“fit”) 3 gains pursued by the health authorities (Table 2): (1) the “rigor (multistakeholder evidence base)” of the AF, the assessment process (promising interrater reliability), and the handbook for assessment organizations, which are in part already reported in scientific publications [21,22,48]; (2) “ISO maintenance,” which refers to the regular ISO review procedures that ensure that the TS remains up to date and potentially evolves to an International Standard; (3) “cross-country recognition/adoption” can be facilitated by the TS, which in turn can increase the number of assessed apps available, optimize resource allocation (the label is effectively a screening), and minimize the duplication of efforts; (4) the “positive effect of the label” on manufacturers’ intent to improve the quality of their app, HCPs’ willingness to prescribe apps, and citizens’ intent to download and ability to choose quality apps [22,48,49]. These effects could be amplified by citizens’ trust in the recommendations of health apps by HCPs (80%) and their expressed need for health authorities to review and rate apps (86%; M Shokralla, MPH, MSc, unpublished data, 2024); (5) the preliminary findings of a comparative analysis of CEN-ISO/TS 82304-2 with European health technology assessment (HTA) frameworks and subsequent alignment [22] show that the handbook has a “significant part of other AFs covered.” For health authorities, this could mean a reduction in the conformity assessment workload because already-labeled apps would only need to be assessed against a limited set of context-specific requirements; and (6) “automated assessments,” which refers to the potential of partially automatizing app assessments, benefiting the quality (rigor), efficiency, affordability, and scalability of assessments and reassessments. Examples include software that can test the app’s accessibility (eg, contrast, addressing color blindness, and readability), privacy compliance (eg, personal data processed and whether the privacy statement includes all mandatory elements [50]), and data security mechanisms (eg, automated security testing) and in general natural language processing to help assess the evidence provided and queries for surveillance purposes.

**Table 2:** Compatibility (or fit) of the gains of adopting an assessment framework (AF) for health apps as perceived by the Catalan and Italian health authorities and the distinct characteristics (gain creators) of the products and services related to CEN-ISO/TS (Comité Européen de Normalisation [European Committee for Standardization]–International Organization for Standardization/Technical Specification) 82304-2:2021 Health software—Part 2: Health and wellness apps—Quality and reliability (the TS).

Gains	Gain creators
Quality apps for use	Rigor (multistakeholder evidence base) ISO maintenance Cross-country recognition/adoption
Prescription and use of apps	Positive effect of the label
Savings and efficiency	Cross-country recognition/adoption Significant part of other AFs covered Automated assessments^a^

^a^Future gain creator planned to be established during the demonstrator phase.

#### Pain Relievers

Twelve current pain relievers and one anticipated pain reliever were identified (Figure 2), which are compatible with (“fit”) 8 pains (Table 3): (1) the “potential of the label for use in app stores,” the common marketplace for health apps. The TS has global applicability (ISO), a label, a scoring mechanism, and scalability potential with the “automated assessments” and “network of ISO-certified assessment organizations.” These distinct characteristics are considered attractive for app stores to rank apps and inform potential users about the quality of (labeled) apps; (2) the “positive effect of the label” on manufacturers’ quality improvement plans and HCPs’ willingness to prescribe apps [22,48,49]; (3) the handbook for app assessment organizations was “aligned with EU-level legislation and values” [22], and the AF was built on the EU Medical Device Regulation and General Data Protection Regulation principles [21]; (4) the TS and Label2Enable project are “EU-initiated and used.” A draft version of the TS was made available to support the creation of COVID-19 apps and referenced in the EU toolbox for COVID-19 contact–tracing apps [51], and the TS is foundational for drafting implementing legislation for labeling wellness applications (that claim interoperability with an electronic health record system) in the European Health Data Space Regulation; (5) the TS is a “CEN-ISO-IEC collaboration”, all 3 bodies are renowned international standardization organizations; (6) the handbook was “informed by a comparative analysis of European HTA frameworks,” that is, the European Network for Health Technology Assessment core model and the Dutch, English, Finnish, French, and German HTA frameworks for health apps [22]; (7) distinctive from other AFs, the AF was “built on 28 standards” [11,21]. This includes the recognized National Institute for Health and Care Excellence evidence standards framework for digital health technologies [52,53], which was used as a foundation for the CEN ISO/TS 82304-2 AF [21]; (8) the “rating matches ISO’s role” to “agree on the best way of doing things, make lives easier, safer and better, enabling trade the world over” [54], enabling authorities to refer to unbiased and standardized assessment results; (9) the “recommendations for reimbursement,” which are the result of a series of Label2Enable workshops for health authorities, HTA bodies, and insurers, with 135 participants from 34 countries [47]; (10) “automated assessments” could help address the need to frequently reassess apps; (11) the visibility of “robust build on the label,” which includes 4 interoperability requirements, is expected to promote manufacturer investments in interoperability; (12) the incorporation of “easy to use on the label” could contribute to addressing inequity in the prescription and use of health apps and promote the continued use of apps [55]; and (13) given the importance of medical societies for HCPs in promoting the prescription and use of apps [56,57], the quality report was “tested with European medical societies” to evaluate and enhance its usefulness for medical societies in providing guidance for HCPs in recommending quality health apps [22]; in addition, the results of mapping the TS with the information needs of cardiologists regarding mobile health solutions, carried out with the European Society of Cardiology, indicated compatibility [58].

**Table 3:** Compatibility (or fit) of the pains in adopting an assessment framework (AF) for health apps as perceived by the Catalan and Italian health authorities and the distinct characteristics (pain relievers) of the products and services related to CEN-ISO/TS (Comité Européen de Normalisation [European Committee for Standardization]–International Organization for Standardization/Technical Specification) 82304-2:2021 Health software—Part 2: Health and wellness apps—Quality and reliability (the TS).

Pains	Pain relievers
Risks of (so many) uncertified apps	Potential of the label for use in app stores Positive effect of the label
Legislation issues	Aligned with EU^a^-level legislation and values EU-initiated and used
Lack of harmonization	CEN-ISO-IEC^b^ collaboration Informed by a comparative analysis of European HTA^c^ frameworks Built on 28 standards EU-initiated and used
Health authority issues	Rating matches ISO’s role
Reimbursement issues	Recommendations for reimbursement
App-related issues^d^	Automated assessments^d^
Data-related issues	Robust build on the label
Citizen and HCP issues	Positive effect of the label Easy to use on the label Tested with European medical societies

^a^EU: European Union.

^b^IEC: International Electrotechnical Commission.

^c^HTA: health technology assessment.

^d^Pain to be addressed and corresponding anticipated pain reliever planned to be established during the demonstrator phase.

## Discussion

### Principal Findings

In this paper, we systematically examined the compatibility of the TS with the needs of 2 health authorities, aiming to further enhance the compatibility of the TS with these needs and, ultimately, reduce the uncertainty of peer authorities in considering the adoption of the TS. The 2 studied health authorities were diverse. FTSS is located in Catalonia, a region in Spain with 8 million citizens, and has an AF for health apps and wearables and an operational assessment process in place. ISS is based in Italy, a country with 59 million citizens, and is working on an AF for telehealth, which includes health apps. We found that despite their diversity, their needs (gains, pains, and jobs) largely overlapped. This suggests that health authorities share common fundamental needs. Differences in needs could be attributed to being a national advisory body in a country currently without an established AF (a focus on legislation issues—Italy) and a regional implementing organization with an AF (a focus on the execution of policy—Catalonia). Both health authorities see a need for, and the benefits of, the uptake of health apps and using a common AF. This confirms the recommendation of the World Health Organization (WHO) to make mHealth evaluation with a common methodology the norm rather than the exception [10]. At the same time, it is apparent that without enabling (EU) legislation and standardization and with multiple authorities involved, it is a challenge to establish an AF. For countries or regions with a small population, arriving at an appropriate (and sufficiently rigorous) AF might not even be feasible, given the costs, capabilities, and limited attractiveness for manufacturers.

When is the compatibility of the TS *sufficient* for health authorities? Osterwalder et al [29] argue that an innovation does not have to address all needs of potential adopters; yet it should satisfy the most essential (unrealized) gains, most extreme (unresolved) pains, and most important (unsatisfied) jobs and preferably be “difficult to copy.” We found that the TS has a range of products and services with distinct characteristics (pain relievers and gain creators) that address in part or in full all but 2 needs of the 2 health authorities studied; these are the jobs “compile policy” and “integrate app data into platform(s)” (Tables 1-3). To what extent these 2 jobs would classify as *important (unsatisfied) jobs*—and as such should be addressed in the future by the TS—needs to be determined in future research. Many of the distinct characteristics (gain creators and pain relievers) are *difficult to copy* (eg, multistakeholder evidence base, international standardization nature, the comprehensiveness of the label, and its development as an EU initiative that has already been applied at the EU level).

Whether all of the distinguished products, services, and their distinct characteristics individually (are perceived to) *sufficiently satisfy* potential adopters’ needs could not be measured conclusively, given the prediffusion stage of the TS at the time of this study. Such measurements and potential further fine-tuning of the compatibility of the TS, which could further reduce uncertainty, are part of the upcoming plans for the demonstrator phase. The aims for this phase include assessing the first 100 apps, developing the aforementioned additional 3 services and 1 gain creator and pain reliever, and testing the health app quality report. We also aim to explore whether a further reduction in the duplication of efforts would increase compatibility and reduce uncertainty. Could, for instance, the need to “arrange easy-to-use platform(s)” for assessed apps be addressed with an EU-level “repository of assessed apps” and EU efforts to materialize the “potential of the label for use in app stores”? Could such strategies increase the rate of adoption of the TS, prevent EU member states (and potentially medical societies) from having to invest individually in such platforms and thus pave the way for the EU-wide adoption of the TS and uptake of health apps in the region? WHO statistics seem to confirm the potential of an EU-level platform, with 77% (39/51) of its European region member states rating the lack of a trustworthy source to access effective apps as a significant barrier to integrating health apps into clinical practice, outnumbering privacy and security concerns (38/51, 75%), patient digital literacy (37/51, 73%), and the lack of evidence on app effectiveness in clinical practice (31/51, 61%) [10].

The health authorities’ jobs can be put in context with the 5 stages of the innovation-decision process in organizations described by Rogers [25]. The first stage is “agenda setting,” when one or more individuals in an organization identify a problem and seek a compatible innovation to solve it. During this stage, the job “compile policy” needs to be carried out. WHO statistics show that 83% (44/53) of its European region member states, including 85% (23/27) of the EU member states, reported having a national digital health policy or strategy. Nevertheless, only 28% (13/47) reported having an entity for mHealth quality oversight, which relates to the second job, “implement” [59].

After agenda setting, the next stages of the innovation-decision process in organizations described by Rogers [25] are “matching” (testing the feasibility of the innovation in solving the organization’s problem), and “redefining/restructuring” (reinventing the innovation to accommodate the organization’s needs and structure). These are related to the identified jobs “arrive at an appropriate AF” and “achieve an efficient assessment process.” Catalonia has already carried out these jobs and published the methodology used to compare the TS to its previous AF and implement the TS, reporting a mere 7% additional context-specific, scope-expanding, and rigor-enhancing requirements [37], similar to reports in the Australian context [60] and as previously suggested [17]. This knowledge could further reduce peer authorities’ uncertainty and inspire their own “matching” and “redefining/restructuring” efforts. The rest of the reported jobs, including “assess apps,” would follow once the first four have been achieved. Going back to the status of the European region reveals that while 83% (44/53) of WHO European region member states reported having a national digital health policy or strategy, only 15% (6/39) reported the evaluation of government-sponsored mHealth (“assess apps”) [10], which is a prerequisite for the ultimate jobs in our list: “integrate app data into platform(s)” and “reimburse/pay for apps.” Although financing schemes for telemedicine are increasingly regularly available [10], the number of reimbursed apps were found to be limited [7] and related value-based pricing frameworks “nonexistent to embryonic” [61].

The low percentages of WHO European region member states having entities for mHealth quality oversight and government-sponsored mHealth evaluation raise questions. Do other member states encounter the same significant challenges (pains) as Catalonia and Italy in satisfactorily arriving at an appropriate AF and assessing health apps? Could that be the reason for not assessing apps? Or could it be that these member states have different needs, perhaps not even perceive, contrary to Catalonia and Italy (and the WHO), that an AF is a much-needed solution? Or do they perhaps perceive other *most essential (unrealized) gains*, *most extreme (unresolved) pains*, and *most important (unsatisfied) jobs*, making an AF less of a priority or other distinct AF characteristics a necessity? Further key informant interviews or analysis of member states' national policies [59] could validate the generalizability of the Catalan and Italian needs or produce alternative customer profiles, perhaps related to the adopter types described by Rogers [25]: innovators, early adopters, early majority, late majority, and laggards. Such an analysis would support future alignment of the TS with the EU region at large; contribute to the development of new services, such as “customized implementation support”; and inform peer authorities’ decision-making on the adoption of the TS. In other words, an analysis of member states’ policies through the lens of the current VPC could potentially support the EU region in scaling the evaluation of mHealth using a common methodology as well as promote the equitable uptake of quality apps. Further “matching” efforts by, or in collaboration with, health authorities, other stakeholders, or relevant projects (eg, European Digital Health Technology Assessment [62] and ASSESS DHT [63]), using their own methodologies or inspiration from Catalonia [37], would reveal whether they similarly have few additional health app quality requirements when integrating CEN-ISO/TS 82304-2 and further evolving the Label2Enable handbook app assessment. These efforts and more multistakeholder positioning statements would further reduce uncertainties.

### Strengths and Limitations

To our knowledge, this study is the first to systematically analyze the compatibility of an existing AF with health authorities’ needs, which has the potential to address the scattered EU landscape, progressing toward a digital single market and improving the uptake of health apps. The timing of the study (prediffusion) exploited the potential to contribute to preadoption compatibility, the effectiveness of the future demonstrator phase, and the rate of adoption of the TS. Our study was based on the perceptions of 3 key informants associated with 2 health authorities in 2 EU countries, each part of a larger landscape of health authorities, each with a focus on health apps as part of a larger scope of digital health technologies (Catalonia: health apps and wearables and Italy: telehealth). The limited number of key informants can be attributed to the prediffusion phase of the TS, that is, the TS was published in 2021, and from 2022 to 2024, the Label2Enable project has cocreated the TS supportive products and services with stakeholders. The 2 health authorities involved—partners in the Label2Enable project—are the first prerelease test users in the EU. The key informants are among the first individuals to evaluate the TS. Such a limited scope requires future validation, and we specifically pledge to extend this analysis to the national policies of the EU member states and further “matching” efforts. The complex process of multistakeholder adoption in the EU and the wider landscape depends on more than compatibility for 2 health authorities in the prediffusion phase. As Catalonia’s FTSS revealed in its paper, a multistakeholder evaluation is recommended as part of adoption decision-making [37]. In other publications, currently or shortly available (all Label2Enable results will be published in Cordis [22] and on the Label2Enable results page [23]), we have addressed other attributes and other key stakeholders, some of whom have produced endorsing positioning statements [58,64]. Theories focusing on the wider ecosystem and AF landscape could be considered to further support adoption strategies. Despite the limitations of this study, the diversity of the 2 health authorities in terms of geographic scope, roles and responsibilities, population size, triggers, the scope of digital health technologies, target pathways and health apps, and the status of the AF, increases the chance of the generalizability of our results.

### Conclusions

Our results suggest compatibility of the TS with the overlapping needs of the health authorities to arrive at an appropriate AF that would allow the uptake of health apps in their health care systems. The perceptions and experiences of health authorities captured in this study through interviews with key informants provide an evidence-based foundation for peer authorities to reduce their uncertainties related to the adoption of an AF, particularly the TS. The study also established the basis to carry out a wider analysis to understand the compatibility of the TS with the needs of other EU member states, which would confirm or fine-tune the TS and its distinct characteristics. To our knowledge, this is the first report to systematically analyze the compatibility of an existing AF with health authorities’ needs, with the potential to address the scattered EU AF landscape, progressing toward a digital single market and improving the uptake of health apps.

## Appendix

Multimedia Appendix 1 The CEN-ISO/TS (Comité Européen de Normalisation [European Committee for Standardization]–International Organization for Standardization/Technical Specification) 82304-2 label.

Multimedia Appendix 2 Interview topic guide.

Multimedia Appendix 3 COREQ (Consolidated Criteria for Reporting Qualitative Research) checklist.

## Abbreviations

AF: assessment framework
CEN: Comité Européen de Normalisation (European Committee for Standardization)
COREQ: Consolidated Criteria for Reporting Qualitative Research
EU: European Union
FTSS: Fundació TIC Salut Social (TIC Salut Social Foundation)
HCP: health care professional
HTA: health technology assessment
ISO: International Organization for Standardization
ISS: Istituto Superiore di Sanità (National Institute of Health)
mHealth: mobile health
TS: Technical Specification
VPC: value proposition canvas
WHO: World Health Organization
