# Supplementary material for: Compatibility of the CEN-ISO/TS 82304-2 Health App Assessment Framework With Catalan and Italian Health Authorities’ Needs: Qualitative Interview Study
Source: JMIR Form Res. 2025 Apr 21;9:e67855. doi: 10.2196/67855 (PMC12053092; doi:10.2196/67855)
Supplement: Multimedia Appendix 1 [file formative_v9i1e67855_app1.docx]

## **Multimedia Appendix 1. The CEN ISO/TS 82304-2 Label**

**Figure MA1. What does the new quality label for health apps show?**
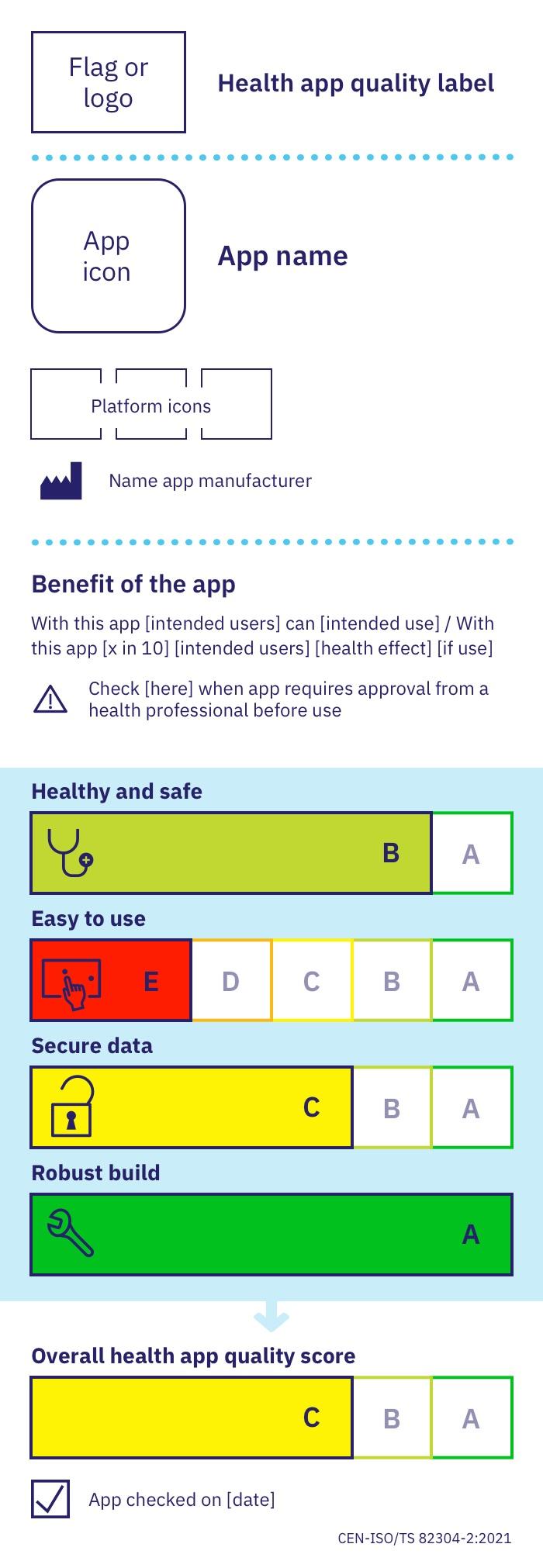


From top to bottom, you see:

The flag or logo of the authority, endorsing the TS.

The App name and icon, followed by the icons of the platforms where the app is available (App store, Google Play store, a website, other), and the name of the manufacturer.

How the health app may benefit you and whether you should consult a health professional before using the app.

The light blue square includes scores for four quality aspects of the health app. These scores can range from A (dark green, the highest score) to E (red, the lowest score).

The “Healthy and safe” score is a sum of 23 quality requirements (4 health requirements, 5 health risks, 2 ethics, 10 health benefit, and 2 societal benefit).

The “Easy to use” score is a sum of 13 quality requirements (5 accessibility and 8 usability).

The “Secure data” score is a sum of 19 quality requirements (8 privacy and 11 security).

Lastly, the “Robust build” score is a sum of 12 quality requirements (8 technical robustness and 4 interoperability).

Underneath the light blue square, the overall quality score (50% Healthy and safe, 15% Easy to use, 25% Secure data, 10% Robust build).

Finally, you see when the app was last assessed.
