# Supplementary material for: Compatibility of the CEN-ISO/TS 82304-2 Health App Assessment Framework With Catalan and Italian Health Authorities’ Needs: Qualitative Interview Study
Source: JMIR Form Res. 2025 Apr 21;9:e67855. doi: 10.2196/67855 (PMC12053092; doi:10.2196/67855)
Supplement: Multimedia Appendix 2 [file formative_v9i1e67855_app2.docx]

## **Multimedia Appendix 2. Interview Topic Guide**

**TOPICS FOR INTERVIEWS AUTHORITIES RESPONSIBLE FOR HEALTH APPS**

**Aim:** To understand how Health apps are integrated in health service provision in your health system, or how are you planning to integrate them. What steps have you taken, are currently taking or will take in the future to make it real? We would also like to explore the role of the app assessment frameworks in this process, and your experience/ openness to using tools like the TS ISO 82304-2 and the accompanying quality label to advance the integration of health apps in health service provision. This step of the research is part of the project activities of WP7 of the Label2Enable project.

**Introduction and context**

1. What type of Health system do you have in your country?

**Task as a national authority (jobs to be done)**

1. What is your country's/region's interest in integrating health apps in health service delivery?
   - What does integrating health apps in your health service mean for your institution?
   - What outcomes are you trying to achieve by integrating health apps in health service delivery?
2. What steps have you taken or are you taking to integrate health apps into your health systems?

- What steps would you like to take in the future to integrate health apps in your health system?
- Do you have a specific policy regarding health apps? If yes, can you share it?
- Does your policy envision Health apps to replace or to be complementary to certain interventions, or certain health services? Do you envision the same thing?
- If complementary or a replacement, In what way?

1. What are the opinions of key stakeholders regarding the integration of health apps in health services?

- Who do you consider your key stakeholders?

1. Do you **assess** the quality of health apps?

- If yes, how did/do you assess the quality of health apps?

1. Do you **communicate** the quality of health apps to your citizens/healthcare professionals?

- If yes, how did/do you communicate the quality of health apps to:

**a**. citizens?

**b**. healthcare professionals?

- How do you evaluate that citizens and healthcare professionals indeed can choose good health apps? Explain more.

1. If you have an assessment framework in place (if not go to the next question):

• What types of metrics of success do you have regarding your assessment framework?

• Which organizations/ teams are involved in assessing and communicating health app quality?

1. Of no assessment framework is yet in place:

• Are you interested in finding a way to assess health apps?

• What frameworks have you explored before?

**Challenges as a national authority (pains):**

1. What are the challenges you have faced or are facing in
   1. integrating health apps?
   2. assessing and communicating the quality of health apps?
2. What will you lose / what is at risk if your “current situation” continues without finding a solution?

**Expected gains as a national authority (gains):**

1. When has your organization done a great job in
2. integrating health apps?
3. assessing and communicating the quality of Health apps?
4. What will you gain if apps are integrated into your health services?

- Who (apart from you) would benefit? (e.g., citizens and healthcare professionals)
  - How would they benefit?

**ISO/TS 82304-2:2021 - Health and wellness apps**

1. What makes you interested in 82304-2?

- Which alternatives have you considered or are you considering?
  - What has been a reason to set them aside / keep them as options?

1. If you decided to go forward with 82304-2 (if not continue to the next question):

- Which steps have you already taken in applying 82304-2? Think for example about comparing, testing, piloting or using.
- What matters most/what makes key stakeholders keen on applying 82304-2?
- Are there reports / evaluations of the application of 82304-2?
- Seeking success factors, learnings, pain relievers, gain creators, etc.

1. If you have not yet decided to go forward with 82304-2:

- What matters most to key stakeholders that could make them keen on applying 82304-2?

1. Who or what is the driving force behind mHealth (policy) in your country / region?

- Would it be good to get them involved in Label2Enable?

1. Is there anything else you wish to add?

Thank you so much for your participation.
