# Supplementary material for: Compatibility of the CEN-ISO/TS 82304-2 Health App Assessment Framework With Catalan and Italian Health Authorities’ Needs: Qualitative Interview Study
Source: JMIR Form Res. 2025 Apr 21;9:e67855. doi: 10.2196/67855 (PMC12053092; doi:10.2196/67855)
Supplement: Multimedia Appendix 3 [file formative_v9i1e67855_app3.docx]

**Multimedia Appendix 3. COREQ (Consolidated Criteria for Reporting Qualitative Research) checklist.**

Tong A, Sainsbury P, Craig J. Consolidated criteria for reporting qualitative research (COREQ): a 32-item checklist for interviews and focus groups. International Journal for Quality in Health Care. 2007. Volume 19, Number 6: pp. 349 – 357

| **No Item** | **Guide questions/description** | **Response** |
| --- | --- | --- |
| **Domain 1: Research team and reflexivity** | | |
| **Personal characteristics** | | |
| 1. Interviewer/ facilitator | Which author/s conducted the interview or focus group? | MV-Q with support from MSh  Reported in section: Authors’ Contributions |
| 2. Credentials | What were the researcher’s credentials? *e.g. PhD, MD* | PhD and PhD candidates  Vast experience in digital health, see www.nell.eu |
| 3. Occupation | What was their occupation at the time of the study? | Senior researcher and junior researcher |
| 4. Gender | Was the researcher male or female? | Female |
| 5. Experience and training | What experience or training did the researcher have? | Qualitative and mixed-methods research, implementation research  Reported in section: Methods |
| **Relationship with participants** | | |
| 6. Relationship established | Was a relationship established prior to study commencement? | The organizations were consortium members of the Label2Enable project  Reported in section: Methods |
| 7. Participant knowledge of the interviewer | What did the participants know about the researcher? *e.g. personal goals, reasons for doing the research* | Participants and interviewers were experts working together in a multidisciplinary project  Reported in section: Methods. |
| 8. Interviewer characteristics | What characteristics were reported about the interviewer/facilitator? *e.g. bias, assumptions, reasons, and interests in the research topic* | Interest in the research topic, and ways to reduce bias issues  Described in sections: Methods and Acknowledgments |
| **Domain 2: Study design** | | |
| **Theoretical framework** | | |
| 9. Methodological orientation and theory | What methodological orientation was stated to underpin the study? *e.g. grounded theory, discourse analysis, ethnography, phenomenology, content analysis* | Theory: Rogers’ Diffusion of Innovations Theory  Stated in sections: Introduction and Methods.  Analysis of interviews: thematic analysis  Stated in section: Methods |
| **Participant selection** | | |
| 10. Sampling | How were participants selected? *e.g. purposive, convenience, consecutive, snowball* | Purposive (key informants)  Stated in section: Methods  Discussed in section: Strengths and Limitations |
| 11. Method of approach | How were participants approached? *e.g. face-to-face, telephone, mail, email* | Via email |
| 12. Sample size | How many participants were in the study? | 2 organizations, 3 interviewees |
| 13. Non-participation | How many people refused to participate or dropped out? Reasons? | None |
| 14. Setting of data collection | Where was the data collected? *e.g. home, clinic, workplace* | The interviews were performed online, and audio recorded  Described in section: Methods |
| 15. Presence of non-participants | Was anyone else present besides the participants and researchers? | No |
| 16. Description of sample | What are the important characteristics of the sample? *e.g. demographic data, date* | Being a key informant from a frontrunner organization in Catalonia and Italy  Key informant was defined in terms of position in the organization, number of years, and knowledge  Stated in section: Methods  Discussed in section: Strengths and Limitations |
| **Data collection** | | |
| 17. Interview guide | Were questions, prompts, and guides provided by the authors? Was it pilot tested? | Yes, and interview guide was used and has been provided as a multimedia appendix  The interview was designed by three researchers with experience in qualitative, mixed-methods and implementation research of digital health  Drafts were shared and discussed with two other researchers to produce the final version  Reported in sections: Methods and Acknowledgments |
| 18. Repeat interviews | Were repeat interviews carried out? If yes, how many? | N/A |
| 19. Audio/visual recording | Did the research use audio or visual recording to collect the data? | Yes, audio recordings  Described in section: Methods |
| 20. Field notes | Were field notes made during and/or after the interview or focus group? | N/A.  However, notes were made during the interview by researchers to highlight discussion points and provide assistance during the analysis |
| 21. Duration | What was the duration of the interviews or focus group? | Between 1.5 and 2 hours  Described in section: Methods |
| 22. Data saturation | Was data saturation discussed? | Thematic saturation was sought across and within cases to achieve a “basis for positioning the innovation so that it will be more acceptable”  Participant validation confirmed we had captured the most relevant aspects  Described in: Methods |
| 23. Transcripts returned | Were transcripts returned to participants for comment and/or correction? | No, instead a participant validation step was carried out separately per authority and lasted between 30 and 60 minutes  Additionally, participants were provided with drafts of the manuscript, including the quotations  Described in sections: Methods and Acknowledgments |
| **Domain 3: Analysis and findings** | | |
| **Data analysis** | | |
| 24. Number of data coders | How many data coders coded the data? | 2 |
| 25. Description of the coding tree | Did the authors provide a description of the coding tree? | The coding tree was deemed unnecessary because the analysis was visualized using the VPC (figure 2) |
| 26. Derivation of themes | Were themes identified in advance or derived from the data? | Six main themes were predefined based on the VPC: gains, pains, jobs, products and services, gain creators and pain relievers  Subthemes were identified inductively  Described in section: Methods |
| 27. Software | What software, if applicable, was used to manage the data? | Atlas.ti Web (v7.9)  Indicated in section: Methods |
| 28. Participant checking | Did participants provide feedback on the findings? | Yes  Described in section: Methods  Discussed in section: Strengths and Limitations |
| **Reporting** | | |
| 29. Quotations presented | Were participant quotations presented to illustrate the themes/findings? Was each quotation identified? *e.g. participant number* | Yes, and quotations were identified and quoted in the main text  Described in section: Results |
| 30. Data and findings consistent | Was there consistency between the data presented and the findings? | Yes |
| 31. Clarity of major themes | Were major themes clearly presented in the findings? | Yes, see VPC, figure 2  Described in section: Results, including tables 1-3 |
| 32. Clarity of minor themes | Is there a description of diverse cases or a discussion of minor themes? | Yes, subthemes were clearly described  Described in section: Results, including tables 1-3 |
